# A Web Tool to Help Counter the Spread of Misinformation and Fake News: Pre-Post Study Among Medical Students to Increase Digital Health Literacy

**DOI:** 10.2196/38377

**Published:** 2023-04-18

**Authors:** Valentina Moretti, Laura Brunelli, Alessandro Conte, Giulia Valdi, Maria Renza Guelfi, Marco Masoni, Filippo Anelli, Luca Arnoldo

**Affiliations:** 1 Dipartimento di Area Medica Università degli Studi di Udine Udine Italy; 2 Accreditamento, Qualità e Rischio Clinico Azienda Sanitaria Universitaria Friuli Centrale Udine Italy; 3 Direzione Medica del Presidio Ospedaliero di San Daniele - Tolmezzo Azienda Sanitaria Universitaria Friuli Centrale San Daniele del Friuli Italy; 4 Dipartimento di Medicina Sperimentale e Clinica Università degli Studi di Firenze Firenze Italy; 5 Federazione Nazionale degli Ordini dei Medici Chirurghi e Odontoiatri Roma Italy

**Keywords:** infodemic, fake news, education, digital health literacy, medical education, medical student, health information, social media, health literacy, online learning, digital education, COVID-19

## Abstract

**Background:**

The COVID-19 pandemic was accompanied by the spread of uncontrolled health information and fake news, which also quickly became an infodemic. Emergency communication is a challenge for public health institutions to engage the public during disease outbreaks. Health professionals need a high level of digital health literacy (DHL) to cope with difficulties; therefore, efforts should be made to address this issue starting from undergraduate medical students.

**Objective:**

The aim of this study was to investigate the DHL skills of Italian medical students and the effectiveness of an informatics course offered by the University of Florence (Italy). This course focuses on assessing the quality of medical information using the “dottoremaeveroche” (DMEVC) web resource offered by the Italian National Federation of Orders of Surgeons and Dentists, and on health information management.

**Methods:**

A pre-post study was conducted at the University of Florence between November and December 2020. First-year medical students participated in a web-based survey before and after attending the informatics course. The DHL level was self-assessed using the eHealth Literacy Scale for Italy (IT-eHEALS) tool and questions about the features and quality of the resources. All responses were rated on a 5-point Likert scale. Change in the perception of skills was assessed using the Wilcoxon test.

**Results:**

A total of 341 students participated in the survey at the beginning of the informatics course (women: n=211, 61.9%; mean age 19.8, SD 2.0) and 217 of them (64.2%) completed the survey at the end of the course. At the first assessment, the DHL level was moderate, with a mean total score of the IT-eHEALS of 2.9 (SD 0.9). Students felt confident about finding health-related information on the internet (mean score of 3.4, SD 1.1), whereas they doubted the usefulness of the information they received (mean score of 2.0, SD 1.0). All scores improved significantly in the second assessment. The overall mean score of the IT-eHEALS significantly increased (*P*<.001) to 4.2 (SD 0.6). The item with the highest score related to recognizing the quality of health information (mean score of 4.5, SD 0.7), whereas confidence in the practical application of the information received remained the lowest (mean of 3.7, SD 1.1) despite improvement. Almost all students (94.5%) valued the DMEVC as an educational tool.

**Conclusions:**

The DMEVC tool was effective in improving medical students’ DHL skills. Effective tools and resources such as the DMEVC website should be used in public health communication to facilitate access to validated evidence and understanding of health recommendations.

## Introduction

Past and current emergencies involving viral outbreaks have demonstrated how difficult and challenging the management of information and communication can be. For example, the rapid evolution of the COVID-19 pandemic led to the proliferation of uncontrolled health information and fake news that “spread faster and easier than the virus,” as noted at the Munich Security Conference on February 15, 2020 [1]. The rapid changes in the pandemic situation and its waves of low-quality scientific news made it difficult for researchers, policy makers, and journalists to constantly adapt public health recommendations to the best available evidence [2]. Conspiracy theories, pseudoscientific health therapies, and fake news about the diagnosis, treatment, prevention, origin, and spread of the virus were widely disseminated and reinforced by mainstream media and social media, in some cases leading to the promotion of risky behaviors [3,4]. Indeed, the terms infodemic and infodemiology are widely known and were defined in the early 2000s [5] after misinformation spread easily with the advent of the world wide web. Since communication is a fundamental element for all public health emergencies, risk communication and misinformation are an integral part of any emergency response [6]. In 2017, the World Health Organization provided a summary of guidance and recommendations for emergency communication that includes the media as part of an integrated communications strategy to protect public health [6], and other key frameworks have been published to address the COVID-19 infodemic [2,7,8].

Evidence suggests that the infodemic has emerged because lack of health literacy (HL) in the population is an underappreciated public health problem [9]. Originally, HL was defined by the US Institute of Medicine in 2000 as “the degree to which individuals have the capacity to obtain, process, and understand basic health information and services needed to make appropriate health decisions” [10]. Later, Norman et al [11] specified a definition of digital health literacy (DHL), focusing on the HL skills required to use electronic devices. Indeed, people with low HL also appeared to have low DHL skills [12]. Because system preparedness interacts with individual preparedness in managing disease outbreaks, DHL, like HL, is considered a key determinant of community and individual health [13,14]. Despite the growing interest in digital health competences in health professions during medical school, related to the potential benefits of the digitization of health care [15-18], the inclusion of this topic in curricula has yet to be addressed [19-21]. Indeed, medical students—as future health professionals directly involved in the delivery and management of health care—should learn to use the best knowledge to guide their practice and help their patients identify healthy beliefs and behaviors [22], and direct them to appropriate internet resources and reliable information. Although there are European educational policy plans and global frameworks [20,21,23-25], the implemented digital education interventions are still heterogeneous and hardly comparable.

To address this problem, an informatics course for medical students specifically focused on DHL has been developed at the University of Florence (Italy). In this course, students use the website “dottoremaeveroche” (DMEVC) [26], a resource created by the Italian National Federation of Orders of Surgeons and Dentists as a type of first-aid communication package for searching terms and problems related to health topics. This website includes a dedicated section, the “Conscious Web Browsing” section, which provides tutorials, downloadable content, and self-administered tests to improve DHL. The aim of this study was to investigate the DHL skills of Italian medical students before and after attending the informatics course with in-depth analysis of the DMEVC web resource.

## Methods

### Description of the Informatics Course

The course is intended for the first year of the Medical School at the University of Florence (Italy). The teachers include authors MRG, with a degree in Computer Science and a PhD in Applied Physiopathology, and MM, a doctor specialized in nuclear medicine. The course is based on an experimental approach both on issues related to the use of information and communication technology in the medical field and on the use of a mix of didactic strategies aimed at enhancing the learning process while allowing the flexible management of a large number of students. Learning outcomes of the course focus on health information management, a fundamental discipline that helps keep up with advances in medical science and combat the rapid obsolescence of medical knowledge. Through general medical information, students acquire the knowledge and skills needed to search the internet and evaluate the quality of medical information. Through scientific information, the students acquire competencies for research in literature databases and are introduced to the conceptual and methodological framework of evidence-based medicine (EBM) as an instrument of medical decision-making. The course is delivered over 6 weeks.

The informatics course is offered as a blended learning experience that combines face-to-face and remote activities in different modalities and at different times [27]. Several previous studies have compared blended and face-to-face learning. In particular, a meta-analysis conducted by the US Department of Education, combining more than 100 studies on the subject mostly drawn from university and health education, showed slightly better performance for students who benefit from blended teaching compared to those who have followed traditional courses [28]. There are many ways to offer blended learning courses. In this informatics course, distance activities are mandatory according to the recommendation based on many studies demonstrating that when optional distance activities are proposed, the percentage of students who carry them out is rather low [29]. The face-to-face activities consist of highly interactive lectures with Mentimeter [30], a freely available student response system [31]. The synchronous sessions are related to learning activities carried out on Moodle, the learning management system of the University of Florence. All students enrolled in the first year of medical school are required to have a Moodle account to enable a two-way communication channel between teachers and students. Lecturers organize the information and communication architecture that is required to optimize the course [32]. Beyond monitoring learning activities, Moodle is used to provide information on the course schedule, including the study of multimedia material, and the start, finish, and delivery of assessment activities. At the same time, students can make observations, pose questions, and offer suggestions that can lead to refining the different phases of the course. Multimedia learning materials available on the web or platform have associated assessment activities to give the students a final grade expressed out of 30. There are three compulsory *e-tivities* (online learning activities): two in the first section and one in the second section of the course. The top grade for each *e-tivity* is 10/30. Students who do not achieve the minimum grade (at least 6/30) in each *e-tivity* must take the oral examination for this part. According to the Italian academic grading system, the maximum overall grade is 30/30 and the minimum overall grade is 18/30.

During the 2020-2021 academic year, the informatics course was held from early November to late December. Due to the constraints of the COVID-19 pandemic, face-to-face classes were replaced by synchronous sessions using Cisco Webex, a software widely used for video conferencing and online meetings. Synchronous sessions were held every 2 weeks and lasted 3 hours each. To avoid student exhaustion, a 10-minute break was taken in the middle of each session. The first synchronic session is used to explain to the students the overall structure of the course, its delivery, and how it will be assessed. At this time, teachers informally ask students if they have taken a similar blended learning course previously. In most cases, almost none of the student answered in the affirmative. In the first lesson, some scenarios are proposed to place the topics of the course in the context of practicing medicine. In addition, the concept of Creative Common License, the technical and legal infrastructure that allows the use and reuse of Open Educational Resources, is introduced, as the use of a massive online open course (MOOC) is included in the course.

The informatics course can then be divided into two sections. The first part deals with web features; how to search the internet; and how to evaluate the quality of medical information in terms of accuracy, trustworthiness, and reliability. The second section deals with Medline and EBM. Most of the topics of the first section are covered by the MOOC titled “The internet and the web information search” (Il Web e la ricerca di informazioni in rete), developed in Italian by MRG and MM, teachers of the informatics course [33]. The MOOC is offered by Federica Web Learning, the main European MOOC platform of Federico II University in Naples (Italy). The course covers the basics of the Internet (TCP/IP protocol and Domain Name System), the characteristics of the web (http and https protocols, HyperText Markup Language, and Uniform Resource Locator), the functioning of search engines, and their evolution from the first to the third generation, with a special emphasis on Google. All students are required to take the MOOC, which awards a badge when they complete the entire course and the self-assessment questions. Finally, students must upload the badge to Moodle. Failure to do so will prevent the electronic learning (e-Learning) platform from administering the assessment test with multiple-choice questions related to the MOOC content.

After retrieving the desired information from a search engine, it is important to evaluate the quality of that information, as one should not assume that the information contained in the top search engine results is accurate and reliable [34]. In addition, the reliability and trustworthiness of internet information are much more susceptible to forgery than printed information, since almost anyone has the ability to develop and share content on the internet. To this end, the DMEVC website is used to teach how to evaluate the quality of medical information on the internet. The global goal of DMEVC is to provide access to reliable and accurate peer-reviewed information on the most frequently asked medical topics. In addition, the website has a section called “Conscious Web Browsing,” which focuses on evaluating the quality of medical information. It consists of three parts: tutorial, interaction, and a downloadable form. The tutorial identifies five criteria for accessing the quality of medical information: authoritativeness of the information source, content, timeliness, transparency, and privacy. For each criterion there is a checklist describing how it should be applied [35]. In the interactive part, the web is used to test students’ ability to evaluate the quality of medical information. Examples of health websites are provided for critical reflection and feedback is provided on the answers given. The final subsection provides a downloadable form that includes questions related to the five criteria previously discussed. The same form is used to assess the knowledge and skills students have acquired to evaluate the quality of medical information. The associated *e-tivity* is to evaluate information from a list of fake websites provided by the teachers. To complete the task, the completed form and a document describing the assessment of the fake website must be uploaded to Moodle. Students’ knowledge growth and their ability to evaluate the quality of medical information were studied in detail using a validated questionnaire, described in the Data Collection section below.

 In the second section of the informatics course, students learn how to use Medline and the basics of EBM. Knowledge of how Medline works is essential for searching the biomedical literature. The use of the Medical Subject Heading (MeSH) database and the difference between keyword and subject searches are explained. Next, teachers focus on EBM, a movement that emerged in the early 1990s with the aim of improving the physician’s decision-making process by considering three main components: scientific evidence, clinical experience, and patient values [36]. The main features of evidence-based practice (EBP) are categorized under the 5As, the difference between background and foreground questions, and the PICO (Patient, Intervention, Comparison, and Outcome) model. In addition, how to extract keywords of interest from PICO and how to enter them into the MeSH database are explained. Keyword searching is indeed extremely important to enable accurate searching of bibliographic sources for students to review and select. An overview of the main types of studies published in the medical literature is provided, following the rules of the evidence pyramid. The difference between systematic and nonsystematic reviews is explained. Finally, the relationship between study types and the clinical question is highlighted to facilitate appropriate medical decision-making for the clinical question under investigation. The *e-tivity* that relates to the second part of the informatics course is an assignment that applies the main principles of EBP. First, students must create a scenario that describes a hypothetical or a real patient with a clinical problem. This approach ensures that the clinical scenario is unique to each student and does not overlap with others. Then, a clinical question must be formulated from the scenario to be transformed according to the PICO model. After identifying keywords, a thematic search must be performed using MeSH terms combined with Boolean operators. From the references found, students must select the most appropriate study according to the evidence pyramid to answer the clinical question (diagnostic, prognostic, therapeutic). In the end, students try to solve the clinical question with the found evidence. Since the students are in the first year of medical school, the accuracy of the clinical answer is not evaluated very strictly. To facilitate the task, an example of a well-done assignment is provided on Moodle. In the final synchronous session, teachers provide feedback on the EBP *e-tivity*. Table 1 summarizes the structure and organization of the course.

Students who are not satisfied with the final grade at the end of the course will be required to take an oral exam on all topics covered in the course. If students are unable to attend the course for any reason, they must create an account on Moodle, complete all of the *e-tivities* detailed on the e-Learning platform, and submit them to the teachers 10 days before the exam. After the *e-tivities* are assessed, students must take an oral exam on the entire course content.

**Table 1:** Structure of the informatics course offered in 2020-2021.

Synchronous sessions		Quizzes and *e-tivities*^a^	Grading
**First section**			
	Introduction to the course, Open and Creative Commons Licensing, Open Educational Resources and MOOC^b^, Introduction to the MOOC *“Il Web e la ricerca di informazioni in rete”*	Using the MOOC, uploading the MOOC badge to Moodle, evaluation test on the MOOC content, completing the pretest questionnaire for data collection	Minimum 6/30; maximum 10/30 (for the evaluation test only)
	Quality of medical information on the internet	Using the “Conscious Web Browsing” [37] from the DMEVC^c^ website, *e*-*tivity* to analyze a medical website; completing the posttest questionnaire for data collection	Minimum 6/30; maximum 10/30 (for the evaluation test only)
**Second section**			
	PubMed, Medline, and Thesaurus MeSH^d^; keyword and topic search; EBM^e^, EBP^f^, PICO^g^ model, evidence pyramid	Writing a paper starting from a clinical scenario from which a clinical question is extracted and transformed into the PICO model, then conducting a thematic search in Medline. Answering the clinical question by selecting the most appropriate type of study according to the evidence pyramid	Minimum 6/30; maximum 10/30
	Feedback on EBP *e-tivity*	—^h^	—

^a^e-tivity: online learning activity; see the text for details of each activity.

^b^MOOC: massive online open course.

^c^DMEVC: *dottoremaeveroche* website.

^d^MeSH: Medical Subject Heading.

^e^EBM: evidence-based medicine.

^f^EPM: evidence-based practice.

^g^PICO: Patient, Intervention, Comparison, and Outcome.

^h^Not applicable.

### Data Collection

Each student participating in the informatics course was asked to self-assess his or her digital literacy in evaluating the quality of health-related information, paying attention to the relevance and reliability of web sources, before and after the guided analysis of the DMEVC web resource and in-depth study of the “Conscious Web Browsing“ section. The tool used for this self-assessment was the eHealth Literacy Scale for Italy (IT-eHEALS), an 8-item self-assessment tool developed by Norman et al [38] to assess eHealth literacy, which was subsequently validated and used in Italy [39]. In addition, questions about the functions of the resource and its quality were added. All responses were scored on a 5-point Likert scale (1=strongly disagree, 5=strongly agree), with higher scores indicating best practices in the use of digital tools for health research. Data collection for the initial evaluation began with a message sent via Moodle to all students asking them to complete the survey on the DMEVC website prior to the start of the course. Participants received information about the aims and methods of the study, as well as assurances of confidentiality and anonymity of their responses. The questionnaire for the second evaluation was given to students after the DMEVC website and the “Conscious Web Browsing” section were explained and the associated *e-tivity* was completed. Variables on sociodemographic characteristics such as age and sex, and internet use for health-related purposes were also collected for each study participant.

### Ethics Considerations

Participation was voluntary, anonymous, and free; thus, formal ethical approval was not required according to European regulation (EU-GDPR). All methods were performed in accordance with relevant guidelines and regulations and with the Declaration of Helsinki and its revised version.

### Data Analysis

Population characteristics are presented as frequency and percentage distributions or as mean (SD) for categorical and continuous variables, respectively. Participants’ responses to each item are presented as frequency, mean, and SD. Item scores were interpreted as follows: mean score<1 as low; ≥1 and <2 as moderate; ≥3 and <4 as intermediate; ≥4 and <5 as high; and 5 as very high. The Wilcoxon rank-sum test was used to assess the relationship between the intervention and the change in responses for each item (significance judged at *P*<.05). All statistical analyses were performed using STATA IC14 software.

## Results

A total of 341 students participated in the study and completed the survey at the beginning of the informatics course (first evaluation). There were 211 (61.9%) female respondents and 130 (38.1%) male respondents. The mean age of the students was 19.8 (SD 2.0) years. Only 8 (2.3%) students were aware of the existence of the DMEVC website prior to taking the course.

**Figure 1:**
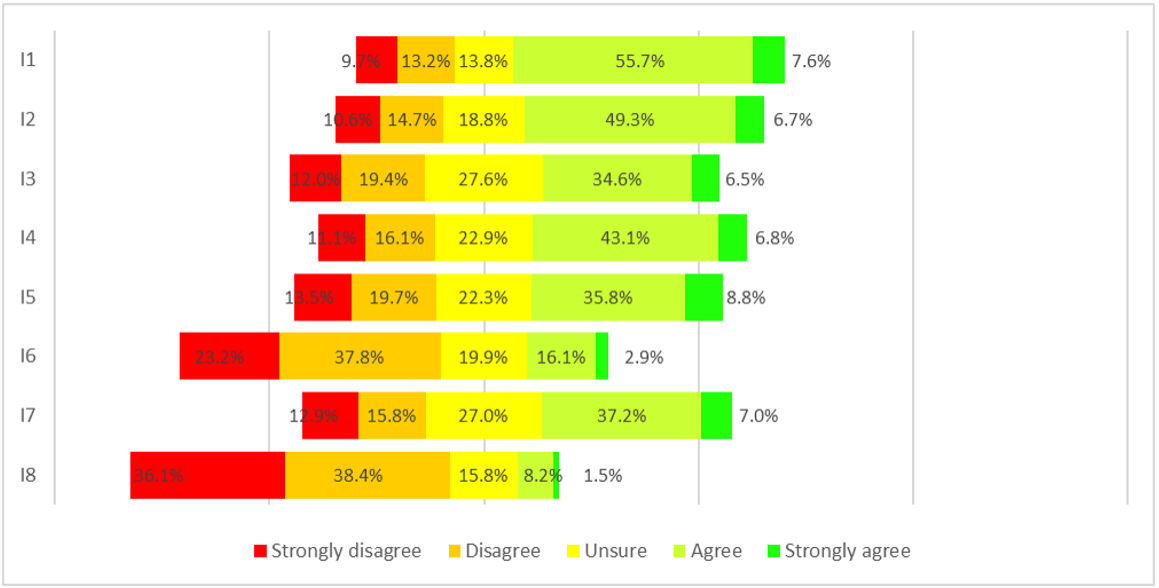
Items response rate at the first evaluation.

At the first evaluation, the mean overall score of the IT-eHEALS was 2.9 (SD 0.9). Among the 314 participants, 216 (63.3%) agreed or strongly agreed about finding helpful health resources on the internet (mean score of 3.4, SD 1.1), and 191 (56.0%) agreed or strongly agreed about how to use the internet to answer health questions (mean 3.3, SD 1.1). Less than half of the participants agreed when it came to what health resources were available on the internet, where to find helpful health resources, how to use health information, and whether to be able to distinguish between and evaluate high-quality and lower-quality health resources. Participants reported difficulty in evaluating health information from the internet, with the most critical item being their perceived confidence in using the information they found to make health decisions; only 33/314 (9.7%) agreed or strongly agreed (mean score of 2.0, SD 1.0). For items characterizing the source, the highest scores were for the importance of authoritative sources, topics, and language used. Participants disagreed with the importance of graphic elements, with 98/314 (28.7%) agreeing or strongly agreeing (mean score of 2.8, SD 1.1), and the presence of sponsors/advertising, with 79/314 (23.2%) agreeing or strongly agreeing (mean score of 2.6, SD 1.2) (Table 2, Figure 1).

**Figure 2:**
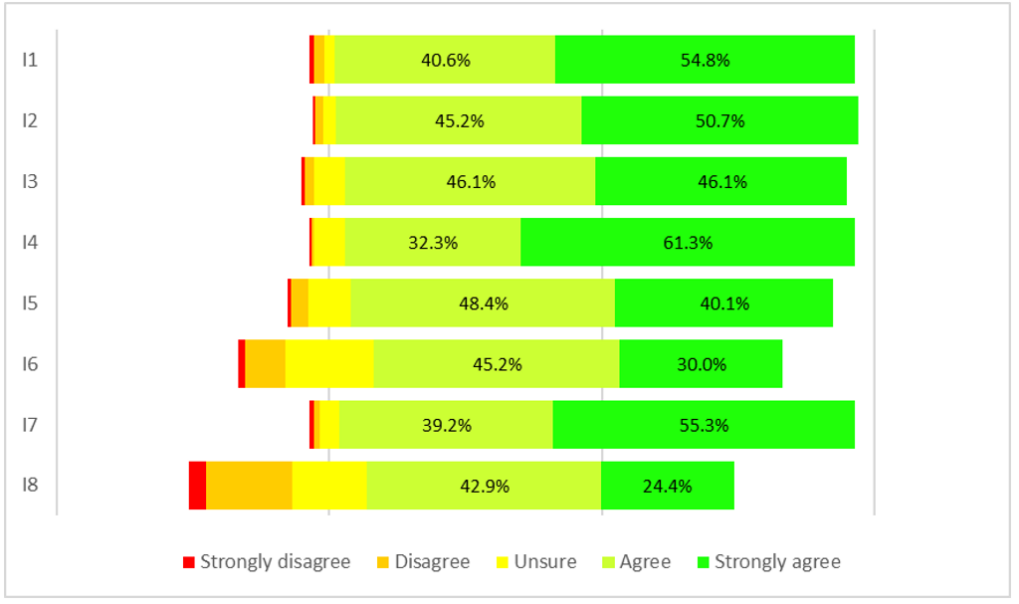
Items response rate at the second evaluation.

A total of 217 (63.6%) students participated in the end-of-course questionnaire (second evaluation). After the explanation of the web resources during the course, 205 (94.5%) students found the section “Conscious Web Browsing” very useful to improve their skills. In the second evaluation, the mean scores of each item improved significantly from those of the first evaluation (Tables 3 and 4; Figure 2). The overall mean score of IT-eHEALS for medical students increased to 4.2 (SD 0.6; *P*<.001), with participants agreeing or strongly agreeing with every item on the survey. More than 90% of students agreed or strongly agreed with where or how to use the internet for health information and what quality information is available on the internet. The most critical items of the IT-eHEALS were those related to the perceived ability to evaluate health information on the internet (163/217 [75.1%] agreed or strongly agreed; mean score of 4.0, SD 0.9; *P*<.001) and trust in the information found (146/217 [67.3%] agreed or strongly agreed; mean score of 3.7, SD 1.1; *P*<.001). Regarding the quality of sources, participants’ opinions improved for all elements and students were only less confident about the importance of graphic elements (143/217 [65.9%] agreed or strongly agreed; mean score of 3.8, SD 1.1).

**Table 2:** Students’ responses at the first evaluation (N=314).

Questionnaire item (I)		Strongly disagree, n (%)	Disagree, n (%)	Undecided, n (%)	Agree, n (%)	Strongly agree, n (%)
**IT-eHEALS^a^**						
	I1: I know how to find helpful health resources on the internet	33 (9.7)	45 (13.2)	47 (13.8)	190 (55.7)	26 (7.6)
	I2: I know how to use the internet to answer my health questions	36 (10.6)	50 (14.7)	64 (18.8)	168 (49.3)	23 (6.7)
	I3: I know what health resources are available on the internet	41 (12.0)	66 (19.4)	94 (27.6)	118 (34.6)	22 (6.5)
	I4: I know where to find helpful health resources on the internet	38 (11.1)	55 (16.1)	78 (22.9)	147 (43.1)	23 (6.8)
	I5: I know how to use the health information I find on the Internet to help me	46 (13.5)	67 (19.7)	76 (22.3)	122 (35.8)	30 (8.8)
	I6: I have the skills I need to evaluate the health resources I find on the internet	79 (23.2)	129 (37.8)	68 (19.9)	55 (16.1)	10 (2.9)
	I7: I can distinguish high-quality from low-quality health resources on the internet	44 (12.9)	54 (15.8)	92 (27.0)	127 (37.2)	24 (7.0)
	I8: I feel confident in using information from the internet to make health decisions	123 (36.1)	131 (38.4)	54 (15.8)	28 (8.2)	5 (1.5)
**Resource elements**						
	I1: Authoritative source	39 (11.4)	10 (2.9)	27 (7.9)	106 (31.1)	159 (46.6)
	I2: Date of the last update	47 (13.8)	21 (6.2)	62 (18.2)	127 (37.2)	84 (24.6)
	I3: Graphic elements	72 (21.1)	40 (11.7)	131 (38.4)	84 (24.6)	14 (4.1)
	I4: Topic	39 (11.4)	3 (0.9)	17 (5.0)	136 (40.0)	146 (42.8)
	I5: Language	39 (11.4)	5 (1.5)	29 (8.5)	158 (46.3)	110 (32.3)
	I6: Transparency	43 (12.6)	33 (9.7)	63 (18.5)	99 (29.0)	103 (30.2)
	I7: Sponsor/advertising	85 (24.9)	62 (18.2)	115 (33.7)	63 (18.5)	16 (4.7)

^a^IT-eHEALS: eHealth Literacy Scale for Italy.

**Table 3:** Students’ responses on the second evaluation (N=217).

Questionnaire item (I)			Strongly disagree, n (%)		Disagree, n (%)		Undecided, n (%)		Agree, n (%)		Strongly agree, n (%)
**IT-eHEALS^a^**											
	I1: I know how to find helpful health resources on the internet	2 (0.9)		4 (1.8)		4 (1.8)		88 (40.6)		119 (54.8)	
	I2: I know how to use the internet to answer my health questions	1 (0.5)		3 (1.4)		5 (2.3)		98 (45.2)		110 (50.7)	
	I3: I know what health resources are available on the internet	1 (0.5)		4 (1.8)		12 (5.5)		100 (46.1)		100 (46.1)	
	I4: I know where to find helpful health resources on the internet	1 (0.5)		1 (0.5)		12 (5.5)		70 (32.3)		133 (61.3)	
	I5: I know how to use the health information I find on the internet to help me	1 (0.5)		7 (3.2)		17 (7.8)		105 (48.4)		87 (40.1)	
	I6: I have the skills I need to evaluate the health resources I find on the internet	3 (1.4)		16 (7.4)		35 (16.1)		98 (45.2)		65 (30.0)	
	I7: I can distinguish high-quality from low-quality health resources on the internet	2 (0.9)		2 (0.9)		8 (3.7)		85 (39.2)		120 (55.3)	
	I8: I feel confident in using information from the internet to make health decisions	7 (3.2)		34 (15.7)		30 (13.8)		93 (42.9)		53 (24.4)	
**Resource elements**											
	I1: Authoritative source	1 (0.5)		1 (0.5)		7 (3.2)		30 (13.8)		178 (82.0)	
	I2: Date of the last update	1 (0.5)		1 (0.5)		9 (4.1)		58 (26.7)		148 (68.2)	
	I3: Graphic elements	9 (4.2)		17 (7.8)		48 (22.1)		79 (36.4)		64 (29.5)	
	I4: Topic	1 (0.5)		1 (0.5)		3 (1.4)		36 (16.6)		176 (81.1)	
	I5: Language	1 (0.5)		4 (1.8)		8 (3.7)		55 (25.3)		149 (68.7)	
	I6: Transparency	2 (0.9)		3 (1.4)		11 (5.1)		35 (16.1)		166 (76.5)	
	I7: Sponsor/advertising	9 (4.2)		3 (1.4)		24 (11.1)		54 (24.9)		127 (58.5)	

^a^IT-eHEALS: eHealth Literacy Scale for Italy.

**Table 4:** Comparison of responses in the first and second evaluations.

Questionnaire items (I)		First evaluation, mean (SD)	Second evaluation, mean (SD)	*P* value
**IT-eHEALS^a^**				
	I1: I know how to find helpful health resources on the internet	3.4 (1.1)	4.5 (0.7)	<.001
	I2: I know how to use the internet to answer my health questions	3.3 (1.1)	4.4 (0.7)	<.001
	I3: I know what health resources are available on the internet	3.0 (1.1)	4.4 (0.7)	<.001
	I4: I know where to find helpful health resources on the internet	3.2 (1.1)	4.5 (0.7)	<.001
	I5: I know how to use the health information I find on the internet to help me	3.1 (1.2)	4.2 (0.8)	<.001
	I6: I have the skills I need to evaluate the health resources I find on the internet	2.4 (1.1)	4.0 (0.9)	<.001
	I7: I can distinguish high-quality from low-quality health resources on the internet	3.1 (1.2)	4.5 (0.7)	<.001
	I8: I feel confident in using information from the Internet to make health decisions	2.0 (1.0)	3.7 (1.1)	<.001
	Overall mean score	2.9 (0.9)	4.2 (0.6)	<.001
**Resource elements**				
	I1: Authoritative source	4.0 (1.3)	4.8 (0.6)	<.001
	I2: Date of the last update	3.5 (1.3)	4.6 (0.6)	<.001
	I3: Graphic elements	2.8 (1.2)	3.8 (1.1)	<.001
	I4: Topic	4.0 (1.2)	4.8 (0.5)	<.001
	I5: Language	3.9 (1.2)	4.6 (0.7)	<.001
	I6: Transparency	3.6 (1.3)	4.7 (0.7)	<.001
	I7: Sponsor/advertising	2.6 (1.2)	4.3 (1.0)	<.001

^a^IT-eHEALS: eHealth Literacy Scale for Italy.

## Discussion

### Principal Findings

The aim of this study was to draw a picture of DHL among Italian medical students and its improvement after a structured educational intervention, whose characteristics were described in detail. The mean average score of the IT-eHEALS at the first evaluation was 2.9 (SD 0.9), suggesting moderate DHL skills. Participants initially found it difficult to find quality health information and the majority of them doubted the usefulness of the information they received on the internet. At the second evaluation, the overall mean IT-eHEALS score increased significantly (mean score of 4.2, SD 0.6; *P*<.001). All scores improved, especially for the items on resource elements of quality. DHL self-assessment showed high confidence in using the internet for medical purposes, whereas uncertainties remained about the practical application of the health information found. The adopted training course showed good results, especially regarding the in-depth analysis of the DMEVC web source. Almost all students had a good understanding of the web resource, demonstrating that the “Conscious Web Browsing” section with its accompanying *e-tivity* was an effective tool to raise awareness of what kind of information is published on the internet and how it is presented. Even though the DMEVC resource led to an effective improvement in students’ DHL, participants seemed to be somewhat aware of the possible unreliability of the information they may find on the internet, which we believe is not a drawback and keeps their vigilance high. Nonetheless, interprofessional collaboration was a fundamental element to provide a comprehensive approach to the topic [16,19,20].

When comparing results with available studies on university students prior to the pandemic, we found that colleagues reported a slightly higher level of DHL, with an overall mean of 3.62 among Jordanian nursing students [40], 3.71 among Korean nursing students [41], and 3.6 among a previous cohort of Italian medical students we studied [42], indicating an intermediate level of confidence in the use of web-based resources for medical purposes. As our survey was conducted during the final phase of the second wave of the COVID-19 pandemic [43], a role played by this stressful period cannot be overlooked. Indeed, the spread of the COVID-19 infodemic may have impaired the perceived ability to find validated information among the misinformation disseminated by the media. Moreover, students’ confidence in the ability to discern reliable information in an era without solid or substantial evidence cannot be overlooked. This hypothesis is supported by a study conducted in Slovenia, which found that the quality of information during the pandemic was a problem even for students with a sufficient level of DHL [14]. Similarly, one-third of German university students during the pandemic reported difficulties in searching for information on health-related topics, and almost half of them doubted the reliability of the web-based results [44]. However, in times of crisis and doubts, as during the pandemic, the ability to use the internet to better inform patients, colleagues, and oneself about the position and recommendations of government and scientific regulatory agencies is much more important.

The skills that future health professionals acquire through the use of this tool could be usefully transferred to patients in the form of recommendations and advice. In addition, the use of DMEVC could also be directly suggested to patients by health professionals as a training tool for critical assessment of resource quality [22]. This could improve patients’ DHL skills and in turn increase their adherence to health-related recommendations. Moreover, this website provides basic and validated information on health topics in a language accessible to nonmedical professionals, and could therefore be considered an official reference communication channel for patients and citizen empowerment.

Finally, it should be noted that the monthly hits on the DMEVC website in 2020 increased compared to those in 2019: +77% in March (start of the pandemic and lockdown measures in Italy), +155% in June, +255% in August, and +364% in October (start of the second wave in Italy). This increase in visibility and use of the website seems to indicate that it was perceived by the public as a useful information source. To continuously raise public awareness and improve DHL among the public, we advocate for broader promotion and continuous updating of this free online educational tool, which would hopefully lead to wider use of the website and increase awareness and DHL. Further improvements to the DMEVC could include tailoring the content based on the user’s DHL level, which should be determined when the user enters the site. In addition, such a resource could be expanded internationally by establishing sister websites for each country that provide up-to-date content in the local language.

### Limitations

DHL was studied using a self-assessment tool that may lead to overestimation of skills, as previously noted in the literature [45,46]. Further objective assessments should be conducted to examine DHL skills and components in depth and to develop specific instructional interventions. Although this study was conducted during the COVID-19 infodemic, students’ information-seeking behavior and awareness of the current public health disposition and situation were not examined. Interestingly, an in-depth analysis of these topics could provide a more comprehensive picture of the impact of the infodemic in the population studied. In addition, considerations must be made about the specific population included in the study and the possible extension of the findings to the general population. For example, a previous European survey of a randomly selected population showed that the level of HL, which directly correlates with DHL [12], is influenced by social differences [47]; accordingly, our medical students, with their high levels of education and health knowledge, may not be representative of the general population.

### Conclusions

Lack of DHL skills may compromise health outcomes as misinformation is amplified by social media and unvalidated web resources. As during the pandemic, the COVID-19 infodemic promoted risky behaviors, some of which compromised public infection control, efforts such as quarantine and isolation measures, protective behaviors, and vaccination adherence. Because DHL skills appear to be inadequate even among medical students, public efforts should aim to provide accessible tools and resources such as the DMEVC website to facilitate access to validated evidence and health recommendations.

## Abbreviations

DHL: digital health literacy
DMEVC: “dottoremaeveroche” website.
EBM: evidence-based medicine
EBP: evidence-based practice
e-Learning: electronic learning
HL: health literacy
IT-eHEALS: eHealth Literacy Scale for Italy
MeSH: Medical Subject Heading
MOOC: massive online open course
PICO: Patient, Intervention, Comparison, and Outcome

## Data Availability

All data generated or analyzed during this study are included in this published article.

## References

[1] (2020). Munich Security Conference. World Health Organization.

[2] Eysenbach G (2020). How to fight an infodemic: the four pillars of infodemic management. J Med Internet Res.

[3] Naeem SB, Bhatti R, Khan A (2021). An exploration of how fake news is taking over social media and putting public health at risk. Health Info Libr J.

[4] Focosi D, Navarro D, Maggi F, Roilides E, Antonelli G (2021). COVID-19 infodemics: the role of mainstream and social media. Clin Microbiol Infect.

[5] Eysenbach G (2002). Infodemiology: the epidemiology of (mis)information. Am J Med.

[6] Emergencies Preparedness, Guidelines Review Committee (2017). Communicating risk in public health emergencies: a WHO guideline for emergency risk communication (ERC) policy and practice. Geneva.

[7] Epidemic and Pandemic Preparedness and Prevention (EPP) WHO Team (2021). WHO competency framework: Building a response workforce to manage infodemics.

[8] Tangcharoensathien V, Calleja N, Nguyen T, Purnat T, D'Agostino M, Garcia-Saiso S, Landry M, Rashidian A, Hamilton C, AbdAllah A, Ghiga I, Hill A, Hougendobler D, van Andel J, Nunn M, Brooks I, Sacco PL, De Domenico M, Mai P, Gruzd A, Alaphilippe A, Briand S (2020). Framework for managing the COVID-19 infodemic: methods and results of an online, crowdsourced WHO technical consultation. J Med Internet Res.

[9] Paakkari L, Okan O (2020). COVID-19: health literacy is an underestimated problem. Lancet Public Health.

[10] Ratzan S, Parker R, Seldon CR, Zorn M, Ratzan SC, Parker RM (2000). Introduction. National Library of Medicine Current Bibliographies in Medicine: Health Literacy. NLM Pub. No. CBM 2000-1.

[11] Norman CD, Skinner HA (2006). eHealth literacy: essential skills for consumer health in a networked world. J Med Internet Res.

[12] Chen X, Hay JL, Waters EA, Kiviniemi MT, Biddle C, Schofield E, Li Y, Kaphingst K, Orom H (2018). Health literacy and use and trust in health information. J Health Commun.

[13] Kickbusch I, Pelikan JM, Apfel F, Tsouros AD (2013). Health literacy: the solid facts.

[14] Vrdelja M, Vrbovšek S, Klopčič V, Dadaczynski K, Okan O (2021). Facing the growing COVID-19 infodemic: digital health literacy and information-seeking behaviour of university students in Slovenia. Int J Environ Res Public Health.

[15] Tudor Car L, Kyaw B, Nannan Panday RS, van der Kleij R, Chavannes N, Majeed A, Car J (2021). Digital health training programs for medical students: scoping review. JMIR Med Educ.

[16] Behrends M, Paulmann V, Koop C, Foadi N, Mikuteit M, Steffens S (2021). Interdisciplinary teaching of digital competencies for undergraduate medical students - experiences of a teaching project by medical informatics and medicine. Stud Health Technol Inform.

[17] Mather CA, Cheng C, Douglas T, Elsworth G, Osborne R (2022). eHealth literacy of Australian undergraduate health profession students: a descriptive study. Int J Environ Res Public Health.

[18] Mesko B, Győrffy Z, Kollár J (2015). Digital literacy in the medical curriculum: a course with social media tools and gamification. JMIR Med Educ.

[19] Poncette A, Glauert DL, Mosch L, Braune K, Balzer F, Back DA (2020). Undergraduate medical competencies in digital health and curricular module development: mixed methods study. J Med Internet Res.

[20] Mosch L, Machleid F, von Maltzahn F, Kaczmarczyk R, Nokhbatolfoghahai F, Balciunas J, Povilonis P, Aktar I (2019). Digital health in the medical curriculum: addressing the needs of the future health workforce. European Medical Students’ Association.

[21] (2020). EU Health Policy Platform - Proposal for a thematic network on digital skills for future-proof doctors (Digital Doc). Europa.

[22] Bigi S, Caporale C, Zagarella R (2020). Politiche del linguaggio in medicina: una prospettiva etica e linguistica.

[23] (2022). Health literacy development for the prevention and control of noncommunicable diseases: volume 3: recommended actions. World Health Organization.

[24] Law N, Woo D, Wong G (2018). A global framework of reference on digital literacy skills for Indicator 4.4.2. Information Paper No. 51. UNESCO.

[25] (2021). Global strategy on digital health 2020-2025. World Health Organization.

[26] dottore, ma è vero che. FNOMCEO.

[27] Guelfi M, Masoni M, Conti A, Gensini G (2011). E-learning in Sanità.

[28] (2010). Evaluation of evidence-based practices in online learning - a meta-analysis and review of online learning studies. US Department of Education.

[29] Hege I, Ropp V, Adler M, Radon K, Mäsch G, Lyon H, Fischer MR (2007). Experiences with different integration strategies of case-based e-learning. Med Teach.

[30] Mentimeter.

[31] Prisco D, Guelfi M, Masoni M, Shtylla J, Federighi P, Ranieri M, Bandini G (2019). Pazienti virtuali nell’insegnamento di Clinica Medica del Corso di Laurea in Medicina e Chirurgia dell’Università di Firenze. Digital Scholarship tra ricerca e didattica.

[32] Trentin G (2008). La sostenibilità didattico-formativa dell'e-learning.

[33] Frederica Web Learning.

[34] Guelfi M, Masoni M, Conti A, Gensini GF (2006). Ricerca e qualità dell'informazione medica disponibile in Internet.

[35] Scheda di valutazione della qualita dell'informazione sanitaria online. dottoremaeveroche.

[36] Evidence-Based Medicine Working Group (1992). Evidence-based medicine. A new approach to teaching the practice of medicine. JAMA.

[37] Sai valutare la qualità dell’informazione sanitaria online?. dottoremaeveroche.

[38] Norman CD, Skinner HA (2006). eHEALS: The eHealth Literacy Scale. J Med Internet Res.

[39] Del Giudice P, Bravo G, Poletto M, De Odorico A, Conte A, Brunelli L, Arnoldo L, Brusaferro S (2018). Correlation between eHealth literacy and health literacy using the eHealth Literacy Scale and real-life experiences in the health sector as a proxy measure of functional health literacy: cross-sectional web-based survey. J Med Internet Res.

[40] Tubaishat A, Habiballah L (2016). eHealth literacy among undergraduate nursing students. Nurse Educ Today.

[41] Kim S, Jeon J (2020). Factors influencing eHealth literacy among Korean nursing students: a cross-sectional study. Nurs Health Sci.

[42] Conte A, Brunelli L, Moretti V, Valdi G, Guelfi M, Masoni M, Anelli F, Parpinel M, Arnoldo L (2023). Can a validated website help improve university students' e-health literacy?. Ann Ig.

[43] COVID-19 integrated surveillance data in Italy. Epicentro.

[44] Dadaczynski K, Okan O, Messer M, Leung A, Rosário R, Darlington E, Rathmann K (2021). Digital health literacy and web-based information-seeking behaviors of university students in Germany during the COVID-19 pandemic: cross-sectional survey study. J Med Internet Res.

[45] Stellefson M, Hanik B, Chaney B, Chaney D, Tennant B, Chavarria E (2011). eHealth literacy among college students: a systematic review with implications for eHealth education. J Med Internet Res.

[46] Ivanitskaya L, O'Boyle Irene, Casey AM (2006). Health information literacy and competencies of information age students: results from the interactive online Research Readiness Self-Assessment (RRSA). J Med Internet Res.

[47] Sørensen K, Pelikan JM, Röthlin F, Ganahl K, Slonska Z, Doyle G, Fullam J, Kondilis B, Agrafiotis D, Uiters E, Falcon M, Mensing M, Tchamov K, van den Broucke S, Brand H, HLS-EU Consortium (2015). Health literacy in Europe: comparative results of the European health literacy survey (HLS-EU). Eur J Public Health.

